# Supplementary material for: The causal relationship between the human gut microbiota and pyogenic arthritis: a Mendelian randomization study
Source: Front Cell Infect Microbiol. 2024 Nov 26;14:1452480. doi: 10.3389/fcimb.2024.1452480 (PMC11629706; doi:10.3389/fcimb.2024.1452480)
Supplement: Supplementary file 1 [file DataSheet1.zip › mendelian test/ebi-a-GCST90027482.csv_forest.pdf]

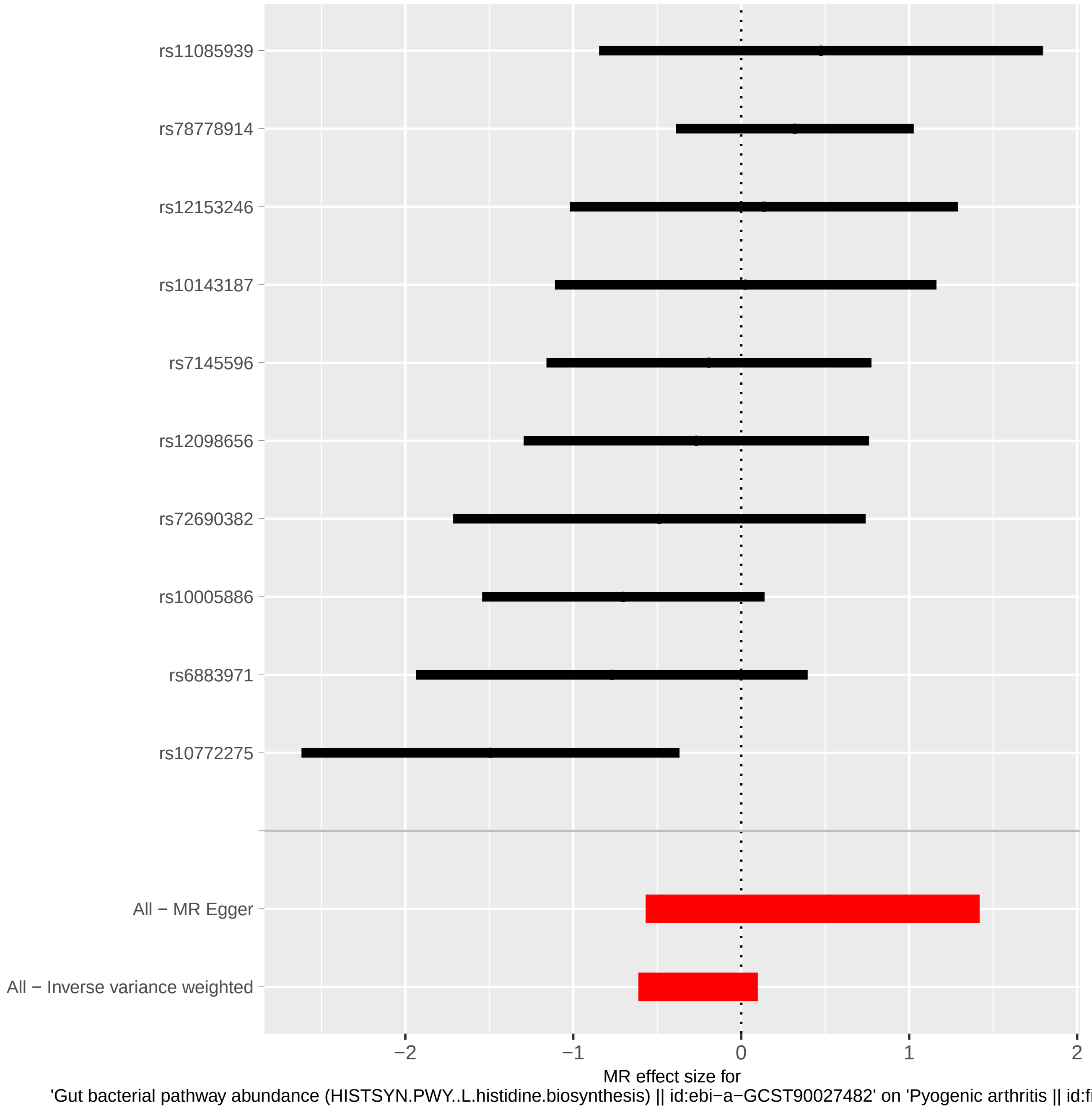
rs11085939
rs78778914
rs12153246
rs10143187
rs7145596
rs12098656
rs72690382
rs10005886
rs6883971
rs10772275
All − MR Egger
All − Inverse variance weighted
−2
−1
0
1
2
MR effect size for
'Gut bacterial pathway abundance (HISTSYN.PWY..L.histidine.biosynthesis) || id:ebi−a−GCST90027482' on 'Pyogenic arthritis || id:fi
